# GmWRKY31 and GmHDL56 Enhances Resistance to *Phytophthora sojae* by Regulating Defense-Related Gene Expression in Soybean

**DOI:** 10.3389/fpls.2017.00781

**Published:** 2017-05-12

**Authors:** Sujie Fan, Lidong Dong, Dan Han, Feng Zhang, Junjiang Wu, Liangyu Jiang, Qun Cheng, Rongpeng Li, Wencheng Lu, Fanshan Meng, Shuzhen Zhang, Pengfei Xu

**Affiliations:** ^1^Soybean Research Institute, Key Laboratory of Soybean Biology of Chinese Education Ministry, Northeast Agricultural UniversityHarbin, China; ^2^Center for Plant Biotechnology, College of Agronomy, Jilin Agricultural UniversityChangchun, China; ^3^First Affiliated Hospital of Harbin Medical UniversityHarbin, China; ^4^Soybean Research Institute, Key Laboratory of Soybean Cultivation of Ministry of Agriculture, Heilongjiang Academy of Agricultural SciencesHarbin, China; ^5^Heihe Branch of Heilongjiang Academy of Agricultural SciencesHeihe, China

**Keywords:** *Glycine max*, GmWRKY31, GmHDL56, *Phytophthora sojae*, response selection

## Abstract

Phytophthora root and stem rot of soybean [*Glycine max* (L.) Merr.] caused by the oomycete *Phytophthora sojae*, is a destructive disease worldwide. The molecular mechanism of the soybean response to *P. sojae* is largely unclear. We report a novel WRKY transcription factor (TF) in soybean, GmWRKY31, in the host response to *P. sojae*. Overexpression and RNA interference analysis demonstrated that GmWRKY31 enhanced resistance to *P. sojae* in transgenic soybean plants. GmWRKY31 was targeted to the nucleus, where it bound to the W-box and acted as an activator of gene transcription. Moreover, we determined that GmWRKY31 physically interacted with GmHDL56, which improved resistance to *P. sojae* in transgenic soybean roots. GmWRKY31 and GmHDL56 shared a common target *GmNPR1* which was induced by *P. sojae*. Overexpression and RNA interference analysis demonstrated that *GmNPR1* enhanced resistance to *P. sojae* in transgenic soybean plants. Several pathogenesis-related (*PR*) genes were constitutively activated, including *GmPR1a*, *GmPR2*, *GmPR3*, *GmPR4*, *GmPR5a*, and *GmPR10*, in soybean plants overexpressing *GmNPR1* transcripts. By contrast, the induction of *PR* genes was compromised in transgenic *GmNPR1*-RNAi lines. Taken together, these findings suggested that the interaction between GmWRKY31 and GmHDL56 enhances resistance to *P. sojae* by regulating defense-related gene expression in soybean.

## Introduction

Phytophthora root and stem rot (PRR), which is caused by the oomycete *Phytophthora sojae*, is one of the most destructive diseases of soybean [*Glycine max* (L.) Merr.] and is responsible for $1–2 billion losses per year worldwide (Tyler, 2007). Since the first identification of PRR in Indiana in 1948, PRR has been observed in all major soybean-growing regions all around the world (Schmitthenner, 1985; Anderson and Buzzell, 1992; Su and Shen, 1993; Jee et al., 1998; Dorrance and Grunwald, 2009). Resistant cultivars carrying major resistance (*R*) genes against *P. sojae* (*Rps* genes) have been a cornerstone for the management of the pathogen for 50 years (Schmitthenner, 1985; Buzzell and Anderson, 1992). *Rps* gene resistance is race-specific, qualitatively inherited and confers an immune type of response to infection by *P. sojae*. However, this qualitative resistance tends to be short-lived because *R*-genes are neutralized by adaptation of *P. sojae* populations (Schmitthenner, 1985).

WRKY transcription factors (TFs) are one of the largest families of TFs in plants (Eulgem et al., 2000; Rushton et al., 2010). WRKY TFs form complex webs to modulate a great number of processes in plants, including senescence, seed development, seed dormancy and germination, and stress responses, particularly in response to biotic and abiotic stresses (Ulker and Somssich, 2004; Zhou et al., 2009; Ren et al., 2010; Rushton et al., 2010; Tao et al., 2011; Jiang et al., 2012; Vanderauwera et al., 2012; Lin et al., 2014). WRKY TFs feature a DNA-binding domain containing the conserved residues WRKYGQK and a novel zinc finger motif (Eulgem et al., 2000). WRKY TFs modulate target gene expression by specifically binding to the *cis*-acting element known as the W-box (C/T)TGAC(T/C) (Rushton et al., 1995; de Pater et al., 1996), which exists in the promoters of many defense-related genes (Maleck et al., 2000; Chen and Chen, 2002; Ulker and Somssich, 2004). GmWRKY27 inhibits the expression of the downstream gene *GmNAC29* by binding to the W-boxes in the promoter region of *GmNAC29*, leading to abiotic stress tolerance (Wang F. et al., 2015), and OsWRKY6 binds directly to WLE1 and W-boxes in the promoters of defense-related genes and regulates pathogen-defense responses (Choi et al., 2015).

Seventy-four WRKY TFs have been reported in Arabidopsis (Pandey and Somssich, 2009; Bakshi and Oelmüller, 2014), and 109 WRKY TFs have been identified in rice, including four with incomplete WRKY domains (Zhang and Wang, 2005). In Arabidopsis, WRKY TFs play a crucial role in the response to pathogen infection (Abuqamar et al., 2006; Journot-Catalino et al., 2006; Li et al., 2006; Xu et al., 2006; Knoth et al., 2007; Lippok et al., 2007; Murray et al., 2007; Zheng et al., 2007; Hu et al., 2008; Mao et al., 2011). Overexpression of *AtWRKY70* leads to the up-regulation of *PR* genes and the down-regulation of *PDF1.2*, leading to enhanced resistance against biotrophic pathogens and increased susceptibility to constitutive necrotrophic pathogens (Li et al., 2004). In addition, mutations of the Arabidopsis *WRKY33* gene enhance susceptibility to the necrotrophic fungal pathogens *Botrytis cinerea* and *Alternaria brassicicola*. By contrast, ectopic over-expression of *WRKY33* increases resistance to these two necrotrophic fungal pathogens (Zheng et al., 2006). Arabidopsis plants overexpressing *AtWRKY28* and *AtWRK75* exhibit enhanced resistance to *Sclerotinia sclerotiorum* (Chen et al., 2013). Several studies have also suggested the importance of specific *OsWRKY*s in the transcriptional regulation of defense-related genes in the rice response to pathogens (Hwang et al., 2011; Lee et al., 2013; Choi et al., 2015). For example, *OsWRKY55* (*OsWRKY31* according to the nomenclature of Wu et al. (2005) is induced by the rice blast fungus, and the transgenic rice plants over-expressing *OsWRKY22* are more resistant to *M. oryzae* (Zhang et al., 2008). *OsWRKY6* plays a role in the pathogen- or salicylic acid (SA)-inducible expression of *OsPR1a*. Indeed, *OsWRKY6* is induced by *Xanthomonas oryzae* pv. *oryzae* (*Xoo*) and SA and activates the *OsPR1a* promoter in rice (Hwang et al., 2011). *OsWRKY22* is a regulator of rice resistance to *M. oryzae* and is also involved in the rice response to non-host barley powdery mildew (Abbruscato et al., 2012). A total of 197 WRKY genes have been identified in soybean [*G. max* (L.) Merr.] (Schmutz et al., 2010), but only four members of the WRKY family in this species have been functionally characterized. Over-expressing *GmWRKY21* or *GmWRKY54* enhances cold tolerance and salt and drought tolerance in Arabidopsis. By contrast, over- expression of *GmWRKY13* leads to increased sensitivity to salt and mannitol stress (Zhou et al., 2008). Transgenic soybean hairy roots overexpressing the *GmWRKY27* gene exhibit enhanced tolerance to drought and salt stresses, whereas *GmWRKY27* RNAi roots exhibit hypersensitivity to these stresses (Wang F. et al., 2015). Although a role of WRKY TFs in abiotic stress responses in soybean has been identified, the potential function of these TFs in biotic stress responses remains unclear.

Homeodomain leucine-zipper (HD-ZIP) TFs, several are known to be rapidly induced in response to altered environmental conditions and to integrate hormonal signals (Brandt et al., 2014). Members of the HD-ZIP proteins play roles related to drought stress and ABA-signaling in different plant species (Dezar et al., 2005; Deng et al., 2006; Agalou et al., 2008). Expression of the HD-ZIP genes AtHB6 and AtHB7 is induced by ABA application or water deficiency (Söderman et al., 1996; Himmelbach et al., 2002; Lechner et al., 2011). Recently, it was shown that both AtHB7 and AtHB12 act to repress the transcription of genes encoding the ABA receptors PYL5 and PYL8 in response to an ABA stimulus (Valdés et al., 2012). In soybean, it has been demonstrated that GmHDL56 and GmHDL57 could interact with the -611 to -451 portion of the *VspB* promoter (Tang et al., 2001). However, the molecular mechanism of HD-ZIP proteins in response to pathogen infection still remains elusive.

In a previous study, a cDNA library of mRNAs encoding expressed sequence tags (ESTs) exhibiting increased expression during *P. sojae* infection was constructed using suppression subtractive hybridization (SSH) from the leaf tissues of the highly resistant soybean ‘Suinong 10’. A novel EST homologous to the WRKY TF *AtWRKY31* (GenBank accession no. NM_118328.3) was significantly induced after infection with *P. sojae* (Xu et al., 2012). In this study, we isolated this WRKY TF, designated *GmWRKY31* (GenBank accession no. XM_003546112.3), from soybean ‘Suinong 10’ and studied the expression and function of this gene. Transgenic soybean plants overexpressing *GmWRKY31* exhibited enhanced resistance to *P. sojae*, whereas RNA interference (RNAi) in transgenic soybean plants increased susceptibility to *P. sojae*. Further analysis indicated that GmWRKY31 physically interacted with GmHDL56 which improved resistance to *P. sojae* in transgenic soybean roots. *GmWRKY31* and *GmHDL56* shared a common target *GmNPR1* (GenBank accession no. NM_001251745.1). *GmWRKY31* and *GmHDL56* coregulated the expression of *GmNPR1* gene, leading to improved soybean resistance to *P. sojae*. These data suggested that GmWRKY31 interacted with GmHDL56 in response to *P. sojae* infection via the activation of *GmNPR1* expression.

## Materials and Methods

### Plant Materials and Stress Treatments

‘Suinong 10,’ a popular soybean cultivar with high resistance against the predominant race 1 of *P. sojae* in Heilongjiang, China (Zhang et al., 2010), was used for gene isolation. Seeds of ‘Suinong 10’ were planted in pots filled with sterile vermiculite in a growth chamber with a 14-h photoperiod (at a light intensity of 350 μmol m^-2^s^-1^) at 22°C/18°C day/night temperatures and relative humidity of 70 ± 10%. Fourteen days after planting, the seedlings at the first-node stage (V1) (Fehr et al., 1971) were used for various treatments.

*Phytophthora sojae* race 1, PSR01, which was isolated from infected soybean plants in Heilongjiang (Zhang et al., 2010), was cultivated at 25°C for 7 days on V8 juice agar in a polystyrene dish. For *P. sojae* treatment, the soybean plants were infected with zoospores according to the method of Ward et al. (1979) with minor modifications for incubation of 48 h. The unifoliate leaves were also harvested at 0, 6, 12, 24, 36, 48, and 72 h after the treatment, immediately frozen in liquid nitrogen, and stored at -80°C until RNA extraction and cDNA analysis.

### Cloning of the Full-Length GmWRKY31 cDNA

The cDNA library of mRNAs encoding ESTs with increased expression during *P. sojae* infection was constructed previously using SSH from the leaf tissues of the highly resistant soybean cultivar ‘Suinong 10’ (Xu et al., 2012). A novel up-regulated EST encoding a putative WRKY TF was identified by searching the phytozome database^1^. In the present study, the full-length cDNA (termed *GmWRKY31*) was isolated by RT-PCR from the cDNA of ‘Suinong 10’ using the primers *GmWRKY31-F*/*R* (Supplementary Table S1). The PCR procedure was performed as follows: 94°C for 5 min, followed by 30 cycles of 94°C for 30 s, 58°C for 30 s, and 72°C for 2 min, with a final extension at 72°C for 10 min. The amplification products were cloned into pMD18-T vector (TaKaRa, Dalian, China) for sequencing.

### Quantitative Real-Time PCR Analysis

Quantitative real-time PCR (qRT-PCR) analysis was performed to determine the transcript abundance of the *GmWRKY31* gene using a SYBR^®^ Green Real Time PCR Master Mix Plus kit according to the manufacturer’s instructions (TOYOBO, Japan) on a CFX96 Touch^TM^ Real-Time PCR Detection System (Bio-Rad, USA). The primers was *GmWRKY31-qF*/*R* (Supplementary Table S1). Total RNA was isolated from soybean leaves using TRIzol reagent (Invitrogen, Shanghai, China) according to the manufacturer’s protocol. Reverse transcription was performed using 1 μg of total RNA and a ReverTra Ace^®^ qPCR RT Kit (TOYOBO, Japan). The PCR protocol was 95°C for 1 min, followed by 40 cycles of 95°C for 15 s, 60°C for 15 s, and 72°C for 45 s. The amplification product was confirmed by melting curve analysis in one-degree intervals from 95 to 60°C. The relative expression value was calculated by the 2^-ΔΔCT^ method using the soybean internal control gene *GmEF1b* (GenBank accession no. NM_001248778) with the primers *GmEF1b-F*/*R* (Supplementary Table S1). Each qRT-PCR was performed on three biological replicates with three technical replicates each.

### Generation and Resistance Identification of Transgenic Plants

To obtain the 35S:*GmWRKY31*-overexpression construct, the coding region of *GmWRKY31* was amplified by RT-PCR using the primers *GmWRKY31-F*/*R* and ligated into the plant overexpression vector pCAMBIA3301^2^ with the *bar* gene as the selective marker. To obtain the 35S:*GmWRKY31*-RNAi silencing construct, a 381 bp fragment of the *GmWRKY31* was amplified by RT-PCR using the primers *GmWRKY31-rF*/*R* and inserted into the RNAi transformation vector pJawohl8 as an inverted-repeat construct with the *pat* gene as the selective marker. To construct the *GmNPR1* overexpression and RNAi vector, the full-length cDNA and a specific cDNA fragment of *GmNPR1* were amplified using the primers *GmNPR1-F*/*R* and *GmNPR1-rF*/*R* and inserted into the pCAMBIA3301 and pJawohl8 vectors, respectively. The overexpression construct and RNAi silencing construct were transferred into *Agrobacterium tumefaciens* LBA4404 via tri-parental mating. The cotyledonary nodes were used as explants for soybean transformation using the *Agrobacterium-mediated* transformation method described by Paz et al. (2004). Homozygous T_2_ plants were identified by glyphosate spraying, PCR amplification, qRT-PCR analysis and southern blot hybridization using a DIG High Prime DNA Labeling and Detection Starter Kit II (Roche, Germany). The full-length cDNA of *GmHDL56* was amplified using the primers *GmHDL56-F*/*R*. The PCR products were inserted into the pCAMBIA3301 vectors. *GmHDL56* transgenic soybean hairy roots were generated by *A. rhizogenes*-mediated transformation according to the method described by Kereszt et al. (2007). The transgenic soybean hairy roots were verified by LibertyLink strip and qRT-PCR analysis. LibertyLink strips (Envirologix, Portland, OR, USA) were used to determinate genetically modified plants containing the phosphinothricin *N*-acetyltransferase protein following the manufacturer’s instruction. All the primers for genotyping and vector construction were listed in Supplementary Table S1.

The transgenic soybean seeds were planted in soil and maintained under greenhouse conditions. For disease resistance analysis, the living cotyledons at the first-node stage (V1) and the trifoliate leaf from the top of each plant at the third-node stage (V3) (Fehr et al., 1971) were treated with *P. sojae* as described by Ward et al. (1979) with minor modifications for the inoculation site and investigated according to the methods described by Dou et al. (2003) with minor modifications for the live plants. The living cotyledons and trifoliate leaf were incubated in a mist chamber at 25°C with 90% relative humidity under a 14-h photoperiod at a light intensity of 350 μmol m^-2^ s^-1^. Non-transformed cotyledons and leaves were used as controls. The disease symptoms on each leaf were observed and photographed using a Nikon D700 camera after inoculation.

### Subcellular Localization of GmWRKY31

The coding sequence of *GmWRKY31* was amplified by RT-PCR using the primers *GmWRKY31-gF*/*R* (Supplementary Table S1). Then, the coding sequence was ligated into the N-terminus of green fluorescence protein (GFP) under the control of the constitutive CaMV35S promoter. The resulting expression plasmid, 35S:GmWRKY31-GFP, was transformed into Arabidopsis protoplasts cells via polyethylene glycol (PEG)-mediated transfection as described by Yoo et al. (2007). The fluorescence signal was imaged using a TCS SP2 confocal spectral microscope imaging system (Leica, Germany). The vector 35S:GFP was used as a control.

### Expression and Purification of Fusion Proteins

The full-length cDNA of *GmWRKY31* was amplified by RT-PCR using the primers *GmWRKY31-yF*/*R* (Supplementary Table S1). The product was inserted at the *BamH*ɪ*/EcoR*ɪ site of the pET29b(+) vector (Novagen, Germany) to generate pET29b(+)-GmWRKY31. The recombinant fusion plasmid was transformed into *Escherichia coli* BL21(DE3) cells. His-tagged proteins were induced with 0.5 mM isopropyl-β-D-thiogalactoside at 37°C for 5 h. The fusion protein was purified at room temperature and quantified according to the pET System Manual (Novagen).

### Electrophoretic Mobility Shift Assay (EMSA)

The DNA binding activity of GmWRKY31 was examined using digoxigenin- ddUTP-labeled double-stranded oligonucleotide W-box or *GmNPR1* promoter DNA N1P (including the W-box domain TTGACC or TTGACT) probes. EMSA was performed as described by Garner and Revzin (1981). The signal was detected by chemiluminescence and recorded on X-ray film (Kodak).

### Transactivation Assays

For transactivation assay, the *GUS* gene in pCAMBIA3301 was replaced by *GmWRKY31* as the effector plasmid. The W-box and sequence from the *GmNPR1* gene promoter was multimerized three times and inserted upstream of the CaMV35S promoter (-42 to +8) containing a TATA box. This construct was inserted into pXGUS-P (Chen et al., 2009) and fused to the *GUS* gene as the reporter plasmid. The transactivation assay was performed by PEG transfection of Arabidopsis protoplasts as described by Yoo et al. (2007). Twenty micrograms of reporter plasmid and 20 μg of effector plasmid or control plasmid (pXGUS-P-35Smini) were co-transfected into 4 × 10^4^ protoplasts. The transfected cells were incubated at 22°C in light for 18–20 h. GUS activity was determined following the methods described by Lu et al. (1998).

### Yeast One-Hybrid Experiment

*GmWRKY31* and GmHDL56 coding regions were amplified and cloned into the pGADT7 prey vector (Clontech)^3^, which created a translational fusion between the GAL4 activation domain and the TF. A specific DNA fragment of the promoter of *GmNPR1* was amplified using the primers N1-F/R (Supplementary Table S1) and cloned into pHIS2 vector at the *EcoR*ɪ/*Sac*ɪ site. Competent yeast cells were prepared according to the Clontech Yeast Protocols Handbook using the Y187 yeast strain. For yeast transformation, 50 μl of competent yeast cells were incubated with 100 ng of pHIS2 bait vector and 100 ng of pGADT7 prey vector, 50 μg of salmon sperm carrier DNA and 0.5 ml of PEG/LiAc solution. Cells were transformed according to the manufacturer’s instructions. Transformations were plated onto SD media (-Leu, -Trp) to select co-transformed cells and incubated at 28°C for 4 days. Transformed yeast cells were subsequently grown in SD media (-Leu, -Trp) liquid media to an OD_600_ of 0.1 and diluted in a 10 × dilution series. From each dilution, 5 μl was spotted on SD media (-Leu, -Trp) and on SD media (-His, -Leu, -Trp) media plates supplemented with 100 mM 3-amino-1, 2, 4-triazole (3AT; Sigma-Aldrich). The plates were then incubated for 3 days at 28°C

### Library Screening

The coding sequence of GmWRKY31 was amplified by RT-PCR using the primers *GmWRKY31-tF* and *GmWRKY31-tR*. Then, the purified PCR product was cloned into the bait vector pGBKT7 (Clontech, USA). The pGBKT7-GmWRKY31 plasmids were transformed into Y_2_H Gold yeast cells. Yeast two-hybrid transformation screens were performed by transforming the pGBKT7-GmWRKY31 expressing strain with the soybean cDNA library, which was constructed using the prey vector pGADT7-rec and mRNA isolated from seedlings of the soybean cultivar ‘Suinong 10’ (Dong et al., 2015) according to the manufacturer’s protocols (Clontech, USA). Cells were screened for growth on SD/-Leu-Trp-His-Ade media for 3–5 days at 30°C. The colonies were transferred to selective media containing X-α-gal (20 μg ml^-1^) and AbA (125 μg ml^-1^). The blue colonies were sequenced and characterized using homology BLAST at NCBI^4^.

### Yeast Two-Hybrid Experiment

The coding region of GmHDL56 was amplified and cloned into pGADT7 (Clontech, USA). The plasmids pGBKT7- GmWRKY31 and pGADT7-GmHDL56 were co-transferred into yeast Y_2_H Gold cells, and interactions were detected by growth on several types of media: SD/-Trp/-Leu media; SD/-Trp/-Leu/-His/-Ade media; and SD/-Trp/-Leu/-His/-Ade/X-α-gal media. Two independent clones for each transformation were tested. The interaction between mammalian pGBKT7-53 and pGADT7-SV40 served as a positive control, whereas the coexpression of pGBKT7-Lam and pGADT7-SV40 served as a negative control (Clontech, USA).

### Bimolecular Fluorescence Complementation (BiFC) Assay

To further confirm the interactions between GmWRKY31 and GmHDL56, a BiFC assay based on yellow fluorescence protein (YFP) was performed. For the constructs, the coding sequence of GmWRKY31 was amplified by RT-PCR using the primers *GmWRKY31-bF*/*R* and cloned into pSAT6-nEYFP-N1, and the coding sequence of GmHDL56 was amplified by RT-PCR using the primers *GmHDL56-bF*/*R* and cloned into pSAT6-cEYFP-N1. The resulting constructs were transformed into Arabidopsis protoplasts cells via polyethylene glycol (PEG)-mediated transfection as described by Yoo et al. (2007). Empty vectors of pSAT6-nEYFP-N1 and pSAT6-cEYFP-N1 were used as controls. Transfected cells were imaged using the TCS SP5 confocal spectral microscope imaging system.

### Pull-Down Assays

Glutathione S-transferase-GmWRKY31 and His-GmHDL56 proteins were separately expressed in *E. coli* BL21(DE3) cells. To purify the recombinant protein, the bacterial cells were pelleted after induction, resuspended in 10 ml of ice-cold 1× binding buffer, and sonicated on ice for 10 min (30-s pulse/min) until the sample was no longer viscous. Following centrifugation at 1,200 × *g* for 15 min at 4°C, the supernatant was harvested and loaded onto a GST- or His-bind Resin column (EMD Millipore, USA). The recombinant protein in the elutes was analyzed by sodium dodecyl sulfate polyacrylamide gel electrophoresis (SDS-PAGE).

Glutathione S-transferase-GmWRKY31 was incubated with GST-sepharose beads for 2 h and washed with 1× binding buffer three times. The beads containing GST-GmWRKY31 were incubated with His-GmHDL56 protein for 1 h. Then, the sepharose beads with associated protein were spun down, washed three times with 1× binding buffer, and boiled in Laemmli sample buffer for 10 min. The pull-down assays were performed at 4°C. The inputs and pull-down pellets of these experiments and the supernatants in the competitive pull-down experiments were subjected to standard SDS-PAGE and immunoblotting using an anti-His antibody.

### Detection of Luciferase Activity in Tobacco Leaf

This assay was performed as previously described (Shang et al., 2010; Song et al., 2013). Briefly, the 1.5 kb promoter sequence of *GmNPR1* (*GmNPR1P*) was cloned using the primers *GmNPR1*-pF/R, and linked to pBI121-LUC (GUS gene of pBI121 was replaced luciferase gene). The reporter construct *GmNPR1P-LUC* and the effector constructs *35S: GmWRKY31* and *35S: GmHDL56* were transformed into *A. tumefaciens* strain GV3101, and transfected into tobacco leaves by agroinfiltration as described previously (Liu et al., 2012). The activity of luciferase was observed using a CCD camera (Berthold Technologies) 72 h after infiltration. All the primers for genotyping and vector construction were listed in Supplementary Table S1.

### SA Measurement

To test whether GmNPR1 could regulate SA accumulation, the content of SA was analyzed in T4 *GmNPR1* transgenic plants. SA was extracted and measured from soybean plant leaves, as described previously by Aboul-Soud et al. (2004). Leaf tissues (0.5 g) were extracted in 1 mL of 90 % methanol following homogenization in liquid nitrogen. 3-Hydroxybenzoic acid (Sigma) was used as an internal standard. The SA extracts were analyzed automatically using a fluorescence detector (excitation at 305 nm and emission at 405 nm) with reversed-phase high-performance liquid chromatography on a Waters 515 system (Waters, Milford, MA, USA) with a C18 column.

## Results

### *GmWRKY31* Expression Is Significantly Induced by *P. sojae*

The full-length cDNA sequence of *GmWRKY31* was cloned from ‘Suinong10’ total RNA by RT-PCR. Sequence analysis indicated that *GmWRKY31* encoded a protein with a deduced polypeptide sequence of 557 amino acids. A WRKY domain was identified at amino acid positions 159–219 (**Supplementary Figure S1A**). Sequence alignment revealed that GmWRKY31 shares 49, 42, 36, and 33% amino acid identity with AtWRKY31, CaWRKY31, GmWRKY23, and GmWRKY6, respectively (**Supplementary Figure S1B**), and the WRKY domains were highly conserved (**Supplementary Figure S1C**).

To study *GmWRKY31* expression, we first examined the accumulation of *GmWRKY31* mRNA in various tissues by qRT-PCR. *GmWRKY31* was constitutively and highly expressed in the leaves, followed by the roots and the stems (**Supplementary Figure S2A**). We further explored the expression pattern of GmWRKY31 in soybean ‘Suinong 10’ during *P. sojae* infection. *GmWRKY31* expression was significantly induced by *P. sojae*, and the accumulation of *GmWRKY31* mRNA reached a peak at 24 h under *P. sojae* stress, followed by a decline (**Supplementary Figure S2B**). The expression of *GmWRKY31* was higher in resistant cultivars (‘Suinong 10,’ ‘Williams 82,’ and ‘Dongnong 1’) than that in susceptible cultivars (‘Dongnong 50,’ ‘Hefeng 35,’ and ‘Hefeng 25’) (**Supplementary Figure S2C**).

### GmWRKY31 Is a W-Box DNA Binding Transcriptional Activator

**FIGURE 1:**
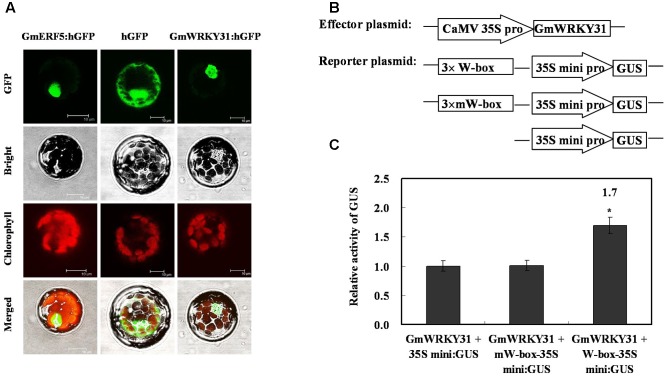
**Subcellular localization of GmWRKY31 protein and transactivation assays of GmWRKY31 to the W-box element.** **(A)** Subcellular localization was investigated in Arabidopsis mesophyll protoplasts under a confocal microscope. The fluorescent distribution of humanized hGFP, the fusion protein GmERF5-hGFP and GmWRKY31-hGFP were observed under white light, UV light, and red light, respectively. Bars, 10 μm. **(B)** Schematic diagram of effector, reporter and internal control plasmid constructs. Effector plasmids encoded GmWRKY31, under the control of the CaMV35S promoter. Reporter plasmids contained three repeats of the wild type or mutant W-box and 35Smini. **(C)** Relative GUS activities in transactivation assays. The effector plasmid encoding GmWRKY31 and the reporter plasmid were co-transfected into Arabidopsis mesophyll protoplasts with internal control plasmid by polyethylene glycol-mediated transformation. The numbers showed the fold increase in GUS activity compared with the vector GmWRKY31+35S mini. Results were averages of three replicates ± standard deviation. Statistically analyzed using Student’s *t*-test (^∗^*P* < 0.05, ^∗∗^*P* < 0.01).

To determine whether GmWRKY31 functions as a TF, we determined the subcellular localization of the GmWRKY31 protein. We transformed the GmWRKY31-GFP construct under the control of the CaMV35S promoter into Arabidopsis mesophyll protoplasts. A strong fluorescent signal derived from GFP alone was observed in the cytoplasm and nuclei, whereas the transformed cells carrying GmWRKY31-GFP displayed a strong green fluorescent signal in the nucleus (**Figure 1A**), just like GmERF5-GFP did (Dong et al., 2015), indicating nuclear localization of GmWRKY31 (**Figure 1A**). We then examined whether GmWRKY31 acts as a transcription regulator in plant cells. We performed a transactivation assay in Arabidopsis mesophyll protoplasts using a reporter gene with three tandem copies of the W-box or mW-box and effector plasmids with GmWRKY31 (**Figure 1B**). As shown in **Figure 1C**, constitutive expression of GmWRKY31 clearly activated the expression of the W-box-driven reporter gene and did not activate the expression of the mW-box-driven reporter gene. The relative GUS activity driven by both the W-box and GmWRKY31 was approximately 1.7 times that of the control (driven by the 35Sm and GmWRKY31), which indicated that GmWRKY31 was able to bind to the W-box and *trans*-activate reporter gene expression in plants. Overall, these results suggest that GmWRKY31 can act as a transcriptional activator in plant cells.

### GmWRKY31 Enhances Resistance to *P. sojae* in Transgenic Soybean Plants

**FIGURE 2:**
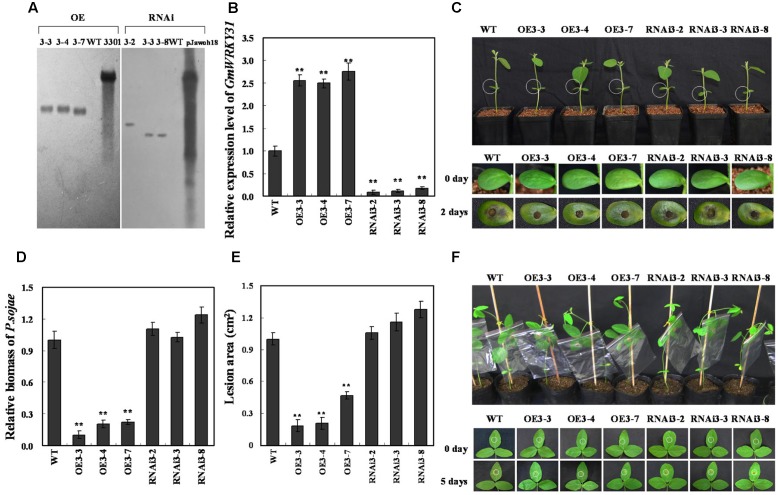
**Response of *GmWRKY31* transgenic soybean plants to *P. sojae*.** **(A)** Southern-blot assay of the T2 *GmWRKY31*-OE, *GmWRKY31*-RNAi and wild-type plants (WT). Twenty micrograms of genomic DNA digested by the restriction enzyme *Hind* III was hybridized with the probe derived from the *bar* gene. **(B)** Quantitative real-time PCR of the T3 *GmWRKY31*-overexpression, *GmWRKY31*-RNAi transgenic soybean plants and WT. *GmEF1b* gene (NM_001248778) was used as an internal control to normalize all data. The experiments were performed on three biological replicates with their respective three technical replicates and statistically analyzed using Student’s *t*-test (^∗^*P* < 0.05, ^∗∗^*P* < 0.01). Bars indicated standard error of the mean. **(C)** Disease symptoms on the living cotyledons of the transgenic lines and WT treated with zoospores of *P. sojae* at 0 and 2 days. **(D)** Accumulation of *P. sojae* biomass in the living cotyledons of the transgenic soybean lines and WT. Transcript levels of *P. sojae TEF1* (EU079791) in infected living cotyledons (2 days) were plotted relative to soybean *GmEF1b* expression levels as determined by qRT-PCR. The experiments were performed on three biological replicates with their respective three technical replicates and statistically analyzed using Student’s *t*-test (^∗^*P* < 0.05, ^∗∗^*P* < 0.01). Bars indicated standard error of the mean. **(E)** Disease symptoms on the living leaves of the transgenic lines and WT treated with a *P. sojae* race 1 inoculum at 0 and 5 days. The experiments were performed on three biological replicates. **(F)** The lesion area of the transgenic lines and WT were detected after 5 days of incubation with *P. sojae*. The experiment was performed on three biological replicates and statistically analyzed using Student’s *t*-test (^∗^*P* < 0.05, ^∗∗^*P* < 0.01). Bars indicated standard error of the mean.

To study the function of *GmWRKY31* in response to *P. sojae*, the plant over-expressing vector 35S:*GmWRKY31* and RNA interference vector 35S:*GmWRKY31*-RNAi were constructed and transformed into soybean plants by the *Agrobacterium*-mediated transformation system. Southern blot results showed that the T2 *GmWRKY31*-overexpressing transgenic lines (*GmWRKY31*-OE) and T2 *GmWRKY31*-RNAi transgenic soybean lines were integrated into the genomes of the three transgenic lines with one copy, respectively (**Figure 2A**). These transgenic lines were developed into T3 transgenic soybean plants. Expression analysis of the transgenic population led to the identification of lines with significantly increased *GmWRKY31* mRNA levels compared with wild-type plants (WT), and expression analysis of T3 *GmWRKY31*-RNAi transgenic soybean plants led to the identification of lines with remarkably reduced *GmWRKY31* mRNA levels compared with WT (**Figure 2B**).

The living cotyledons of those transgenic soybean plants were selected to investigate resistance to *P. sojae*. After 2 days of incubation with zoospores of *P. sojae*, a remarkable difference in the development of disease symptoms was observed (**Figure 2C**). In *GmWRKY31*-RNAi lines, the cotyledons became soft, emitted a foul odor and exhibited clear water-soaked lesions compared with those of the *GmWRKY31*-OE lines, and there were nearly no visible lesions in *GmWRKY31*-OE lines and the cotyledons keep hard compared with those of the *GmWRKY31*-RNAi lines or WT. Meanwhile, tthe relative biomass of *P. sojae* in infected living cotyledons after 2 days of incubation with zoospores of *P. sojae* was also analyzed. The biomass of *P. sojae* based on the transcript level of the *P. sojae TEF1* gene (GenBank accession no. EU079791) was significantly (*P* < 0.01) lower in *GmWRKY31*-OE lines than that in WT, and it was higher in *GmWRKY31*-RNAi lines than that in WT but did not reach a significant level (**Figure 2D**). Similar results were obtained after 5 days of incubation with *P. sojae*, and the living leaves of the WT and *GmWRKY31*-RNAi soybean plants exhibited clear, large lesions compared with those of the *GmWRKY31*-OE lines (**Figure 2E**). The lesion area of the *GmWRKY31*-OE lines was significantly (*P* < 0.01) smaller than that of WT soybean plants after 5 days of incubation with *P. sojae* (**Figure 2F**). These results indicate that overexpression of *GmWRKY31* in soybean plants improves resistance to *P. sojae* and that *GmWRKY31*-RNAi transgenic soybean plants have increased susceptibility to *P. sojae*.

### GmWRKY31 Interacts with GmHDL56

**FIGURE 3:**
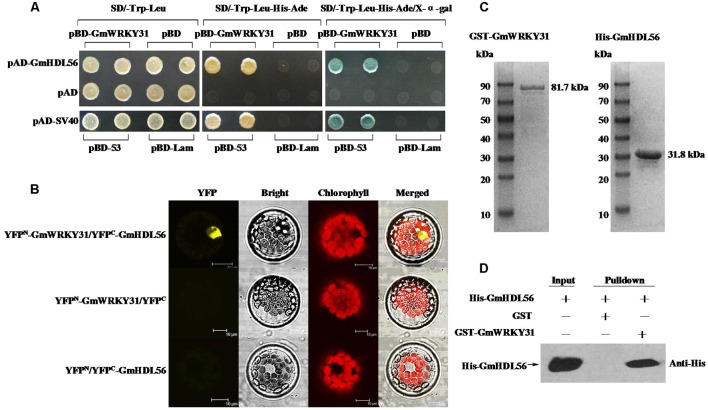
**Interaction of GmWRKY31 with GmHDL56 *in vitro* and *in vivo*.** **(A)** Analysis of interactions between GmWRKY31 and GmHDL56 protein in yeast cells. The yeast cells of strain Y_2_H harboring pBD-GmWRKY31 and pAD-GmHDL56 plasmid combinations were grown on either SD/-Trp/-Leu media or SD/-Trp/-Leu/-His/-Ade media, followed by β-galactosidase assay (SD/-Trp/-Leu/-His/-Ade/X-α-gal media). **(B)** Bimolecular fluorescence complementation (BiFC) analysis of interaction between GmWRKY31 and GmHDL56 in *Arabidopsis* protoplast cells. The plasmid combinations are indicated on top. The fluorescence of YFP was observed by confocal laser microscopy 16 h after transfection. Bars = 10 μm. **(C)** SDS-PAGE analysis of the purified recombinant proteins GST-GmWRKY31 and His-GmHDL56 using GST- or His-Bind Kits. **(D)** Pull-down assay of GmWRKY31 interaction with GmHDL56. His-GmHDL56 protein was incubated with immobilized GST or GST-GmWRKY31 proteins, and immunoprecipitated fractions were detected by anti-His antibody.

To determine which protein is responsible for the interaction with GmWRKY31, we performed yeast two-hybrid screening to identify WRKY31-interacting partners using a soybean cDNA library (Dong et al., 2015). We identified 19 putative GmWRKY31-interacting proteins (Supplementary Table S2), of which a cDNA corresponding to the homeodomain-leucine zipper transcript factor GmHDL56 (LOC100796213) was selected for further study. Yeast two-hybrid assays revealed that GmWRKY31 interacts strongly with GmHDL56 (**Figure 3A**). Bimolecular fluorescence complementation (BiFC) analyses revealed that GmWRKY31 interacts with GmHDL56 in the nuclei of Arabidopsis cells (**Figure 3B**). Consistent with the results of the BiFC assay, an *in vitro* glutathione S-transferase (GST) pull-down assay was performed using a recombinant N-terminal GST-tagged GmWRKY31 protein and a recombinant C-terminal His-tagged GmHDL56 protein (**Figure 3C**). As shown in **Figure 3D**, the His-tagged GmHDL56 protein was pulled down by GST-GmWRKY31 but not by GST alone, indicating that GmWRKY31 interacts with GmHDL56 *in vitro*. These results suggest that GmWRKY31 directly interacts with GmHDL56.

### GmHDL56 Enhances Resistance to *P. sojae* in Transgenic Soybean Hairy Roots

**FIGURE 4:**
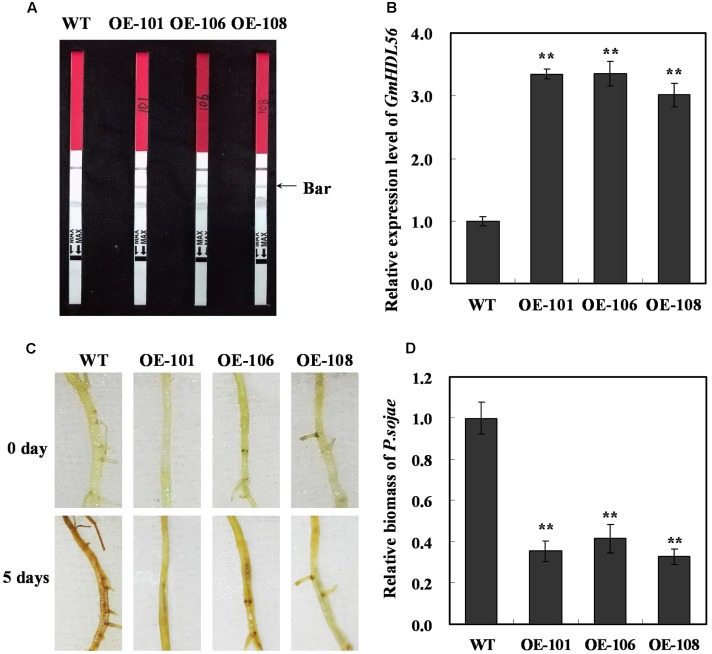
**GmHDL56 enhances resistance to *P. sojae* in transgenic soybean hairy roots.** **(A)** Transgenic soybean plants were tested using Liberty Link strips. **(B)** Quantitative real-time PCR of transgenic soybean hairy roots overexpressing *GmHDL56* and WT. *GmEF1b* gene (NM_001248778) was used as an internal control to normalize all the data. The experiments were performed on three biological replicates with their respective three technical replicates and statistically analyzed using Student’s *t*-test (^∗^*P* < 0.05, ^∗∗^*P* < 0.01). Bars indicate standard error of the mean. **(C)** Disease symptoms on the hairy roots of the transgenic lines and WT treated with zoospores of *P. sojae* at 0 and 5 days. **(D)** Accumulation of *P. sojae* biomass in the hairy roots of the transgenic soybean lines and WT. Transcript levels of *P. sojae TEF1* (EU079791) in infected hairy roots (5 days) were plotted relative to soybean *GmEF1b* expression levels as determined by qRT-PCR. The experiments were performed on three biological replicates with their respective three technical replicates and statistically analyzed using Student’s *t*-test (^∗^*P* < 0.05, ^∗∗^*P* < 0.01). Bars indicated standard error of the mean.

The interaction between GmWRKY31 and GmHDL56 raised the question of whether GmHDL56 was also required for *P. sojae* responses in soybean. To this end, we examined the phenotype of GmHDL56 in response to *P. sojae* infection. The plant over-expressing vector *35S:GmHDL56* was constructed and transformed into soybean hairy roots by high-efficiency *Agrobacterium rhizogenes*-mediated transformation (Kereszt et al., 2007). The transgenic soybean hairy roots were tested using LibertyLink strips (**Figure 4A**) and qRT-PCR (**Figure 4B**). Three overexpression hairy roots (OE101, OE106, and OE108) were selected to investigate resistance to *P. sojae*. After 5 days of incubation with zoospores of *P. sojae*, three *GmHDL56*-overexpressing lines (OE101, OE106, and OE108) displayed significantly enhanced resistance compared with the wild-type plants (**Figure 4C**). Moreover, we analyzed the relative biomass of *P. sojae* in soybean root based on the transcript level of the *P. sojae TEF1* gene. The results indicated that the *P. sojae* biomass was lower in *GmHDL56*-overexpressing lines than that in wild-type plants (**Figure 4D**). These data suggest that GmHDL56 positively regulates the soybean defense in response to *P. sojae* infection.

### *GmWRKY31* Positively Regulates *GmNPR1* Expression via Directly Binds to the W-Box on the Promoter of *GmNPR1*

**FIGURE 5:**
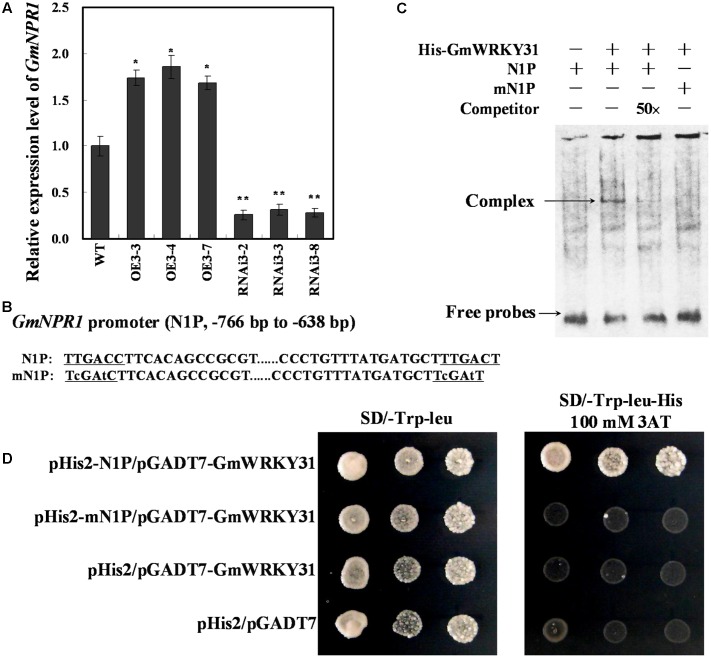
**GmWRKY31 positively regulate *GmNPR1* expression and bound to the W-box on the –766 to –638 region of the *GmNPR1* promoter.** **(A)** Relative expression level of *GmNPR1* gene determined by qRT-PCR. *GmEF1b* gene (NM_001248778) was used as an internal control to normalize all data. The experiment was performed on three biological replicates with their respective three technical replicates and statistically analyzed using Student’s *t*-test (^∗^*P* < 0.05, ^∗∗^*P* < 0.01). Bars indicated standard error of the mean. **(B)** Nucleotide sequences of the N1P and mN1P probes. **(C)** EMSA analysis of binding of GmWRKY31 protein to the W-box. A 129 bp *GmNPR1* promoter fragment containing the W-box or mW-box was labeled with digoxigenin-ddUTP and used as probe. An unlabeled probe was used as a cold competitor. **(D)** The result of yeast-one-hybrid assay for interaction between GmWRKY31 and W-box. The –766 to –638 region of the *GmNPR1* promoter containing two W-box (N1P) or two mutated W-box (mN1P) were used as bait. Yeast cells were dropped in 10-fold dilutions onto selective media (SD/-Leu-Trp-His) containing 100 mM 3-AT (3-amino-1, 2, 4-triazole). pHIS2 co-transformed with pGAD-GmWRKY31 was used as negative control. Each transformation used three different colonies of each pairwise interaction test.

WRKY proteins act upstream of *NPR1* and positively regulate its expression during the activation of the plant defense response (Yu et al., 2001; Liu et al., 2005; Choi et al., 2015). We previously reported that *GmNPR1* was induced after infection with the oomycete *P. sojae* (Xu et al., 2012). To determine whether *GmNPR1* is involved in *GmWRKY31*-overexpression-mediated defense pathways in soybean, we analyzed *GmNPR1* mRNA levels in *GmWRKY31* transgenic soybean plants by qRT-PCR. *GmNPR1* was highly induced in *GmWRKY31*-overexpressing lines and repressed in *GmWRKY31*-RNAi lines (**Figure 5A**), which suggested that *GmWRKY31* might be directly or indirectly involved in the regulation of *GmNPR1*. We then analyzed the *GmNPR1* genomic sequence and identified two W-box elements in the -766 to -638 region of the *GmNPR1* promoter (N1P) (**Figure 5B**). To determine whether the GmWRKY31 protein directly binds to these element sequences, EMSAs using DNA fragments corresponding to the N1P region and the GmWRKY31 protein produced in *E. coli* was performed. As shown in **Figure 5C**, the recombinant GmWRKY31 bound only to N1P, and not mN1P. In addition, increasing molar excesses of unlabeled N1P fragment (competitor) inhibited binding (**Figure 5C**). These results indicate that GmWRKY31 specifically binds to the -766 to -638 region of the *GmNPR1* promoter *in vitro*.

To further confirm that GmWRKY31 binds to the -766 to -638 region of the *GmNPR1* promoter *in vivo*, a yeast one-hybrid assay was performed. As shown in **Figure 5D**, GmWRKY31 bound to N1P but not mN1P in the Y1H assay. These results indicate that GmWRKY31 directly binds to the -766 to -638 region of the *GmNPR1* promoter *in vivo*.

### GmHDL56 and GmWRKY31 Coregulates *GmNPR1* Expression

**FIGURE 6:**
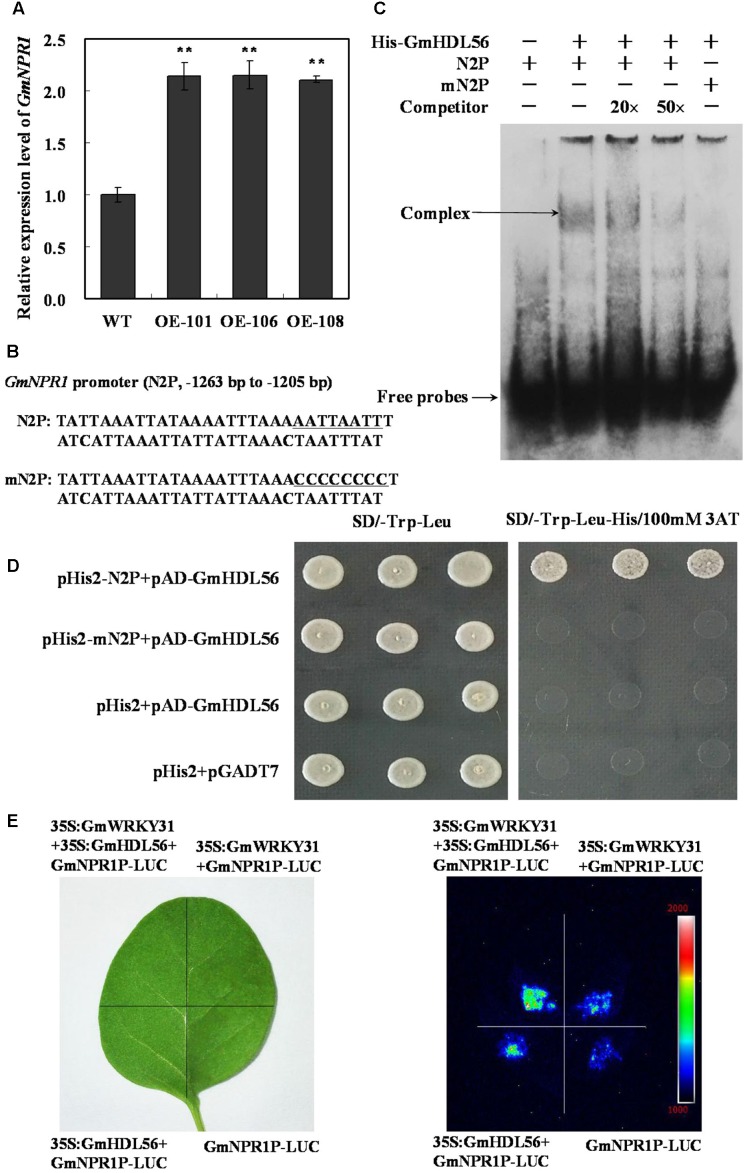
**GmWRKY31 and GmHDL56 coregulated *GmNPR1* expression.** **(A)** Relative expression level of *GmNPR1* gene determined by qRT-PCR. *GmEF1b* gene (NM_001248778) was used as an internal control to normalize all the data. The experiments were performed on three biological replicates with their respective three technical replicates and statistically analyzed using Student’s *t*-test (^∗^*P* < 0.05, ^∗∗^*P* < 0.01). Bars indicate standard error of the mean. **(B)** Nucleotide sequences of the N2P and mN2P probes. **(C)** EMSA analysis of binding of GmHDL56 protein to the ATTAATTA element present at –1263 to –1205 region of the *GmNPR1* promoter. A 59 bp *GmNPR1* promoter fragment containing the element was labeled with digoxigenin-ddUTP and used as probe. An unlabeled probe was used as a cold competitor. **(D)** The result of yeast-one-hybrid assay for interaction between GmHDL56 and ATTAATTA element. The –1263 to –1205 region of the *GmNPR1* promoter containing ATTAATTA element (N2P) or mutated W-box (mN2P) were used as bait. Yeast cells were dropped in 10-fold dilutions onto selective media (SD/-Leu-Trp-His) containing 100 mM 3-AT (3-amino-1, 2, 4-triazole). pHIS2 co-transformed with pGAD-GmHDL56 was used as negative control. Each transformation used three different colonies of each pairwise interaction test. **(E)** GmWRKY31 and Gm56HDL activate GmNPR1 promoter activity in tobacco leaves. *A. tumefaciens* GV3101 strains harboring GmNPR1P-LUC and 35S: GmWRKY31 and/or GmHDL56 were transfected into tobacco leaves. Luciferase imaging was performed 72 h after injection.

To determine whether GmHDL56 regulates *GmNPR1* gene expression, we analyzed *GmNPR1* mRNA levels in the *GmHDL56* transgenic hairy roots. RT-qPCR results showed that *GmNPR1* was highly induced in *GmHDL56* overexpression hairy roots (**Figure 6A**). GmHDL56 could bind to the phosphate response domain ATTAATTA of the soybean VspB tripartite promoter (Tang et al., 2001). To determine whether GmHDL56 directly binds to *GmNPR1* promoter, we analyzed the nucleotide sequence of *GmNPR1* promoter and identified an ATTAATTA element in the -1263 to -1205 region of the *GmNPR1* promoter (N2P) (**Figure 6B**). To determine whether GmHDL56 directly binds to the element sequences, we performed EMSA using DNA fragments corresponding to the N2P region. As shown in **Figure 6C**, recombinant GmHDL56 bound to N2P but not mN2P. In addition, increasing molar excesses of the unlabeled N2P fragment (competitor) inhibited the binding. These results indicated that GmHDL56 binds specifically to the ATTAATTA element (-1263 to -1205) present in the promoter of region of *GmNPR1 in vitro*. To further confirm that GmHDL56 binds to the -1263 to -1205 region of the *GmNPR1* promoter *in vivo*, a yeast one-hybrid assay was performed. As shown in **Figure 6D**, GmHDL56 bound to N2P but not mN2P in the Y1H assay. These results indicated that GmHDL56 directly binds to the -1263 to -1205 region of the *GmNPR1* promoter *in vivo*.

To learn how the interaction between GmWRKY31 and GmHDL56 regulates the expression of *GmNPR1* gene. We examined whether the GmNPR1 promoter activity was activated by the two proteins using the luciferase expression system *in vivo*. The luciferase under the control of the GmNPR1 promoter was injected into tobacco leaves simultaneously with *35S:GmWRKY31*and/or*35S:GmHDL56*. Our results showed that both GmWRKY31 and GmHDL56 improved GmNPR1 promoter activity, and the activation was stronger when the two proteins were simultaneously expressed (**Figure 6E**). The above findings suggest that both GmWRKY31 and GmHDL56 are transcriptional activators of *GmNPR1*, and they work together to improve transcriptional activity.

### *GmNPR1* Enhances Resistance to *P. sojae* and Positively Regulated the Expression of the Pathogenesis-Related (*PR*) Genes in Transgenic Soybean Plants

To evaluate the responsiveness of *GmNPR1* to *P. sojae*, qRT-PCR was performed to determine the expression patterns of *GmNPR1* in ‘Suinong 10’ plants. The examination of tissue-specific transcript abundance in ‘Suinong 10’ demonstrated that *GmNPR1* was constitutively and highly expressed in the roots, followed by the leaves and stems (**Supplementary Figure S3A**). Under *P. sojae* stress, *GmNPR1* mRNA rapidly increased and reached a maximum level at 72 h after the treatment (**Supplementary Figure S3B**). The expression of *GmNPR1* was higher in resistant cultivars (‘Suinong 10,’ ‘Williams 82,’ ‘Dongnong 1’) than that that in susceptible cultivars (‘Dongnong 50,’ ‘Hefeng 35,’ and ‘Hefeng 25’) (**Supplementary Figure S3C**).

**FIGURE 7:**
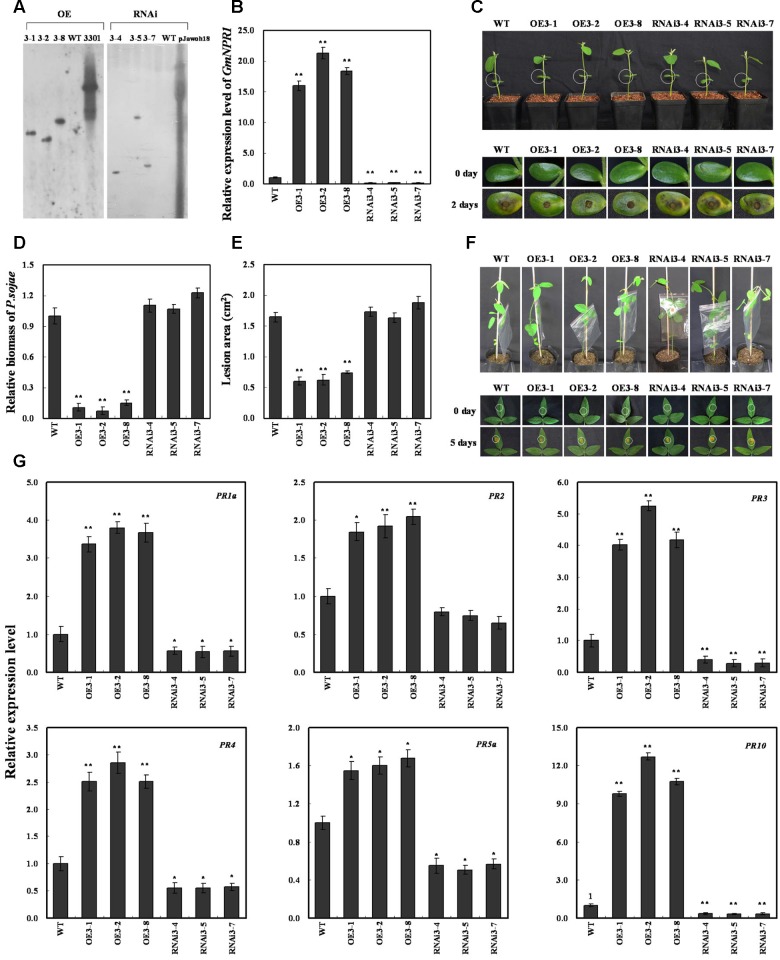
**Response of *GmNPR1* to *P. sojae* in transgenic soybean plants.** **(A)** Southern-blot assay of the T2 *Gm NPR1*-OE, *Gm NPR1*-RNAi and wild-type plants (WT). Twenty micrograms of genomic DNA digested by the restriction enzyme *Hind* III was hybridized with the probe derived from the *bar* gene. **(B)** Quantitative real-time PCR of the T3 *GmNPR1*-overexpression, *GmNPR1*-RNAi transgenic soybean plants and WT. *GmEF1b* gene (NM_001248778) was used as an internal control to normalize all data. The experiments were performed on three biological replicates with their respective three technical replicates and statistically analyzed using Student’s *t*-test (^∗^*P* < 0.05, ^∗∗^*P* < 0.01). Bars indicate standard error of the mean. **(C)** Disease symptoms on the living cotyledons of the transgenic lines and WT treated with zoospores of *P. sojae* at 0 and 2 days. The experiments were performed on three biological replicates with their respective three technical replicates and statistically analyzed using Student’s *t*-test (^∗^*P* < 0.05, ^∗∗^*P* < 0.01). Bars indicate standard error of the mean. **(D)** Accumulation of *P. sojae* biomass in the living cotyledons of the transgenic soybean lines and WT. Transcript levels of *P. sojae TEF1* (EU079791) in infected living cotyledons (2 days) were plotted relative to soybean *GmEF1b* expression levels as determined by qRT-PCR. The experiments were performed on three biological replicates with their respective three technical replicates and statistically analyzed using Student’s *t*-test (^∗^*P* < 0.05, ^∗∗^*P* < 0.01). Bars indicate standard error of the mean. **(E)** Disease symptoms on the living leaves of the transgenic lines and WT treated with a *P. sojae* race 1 inoculum at 0 and 5 days. The experiments were performed on three biological replicates with their respective three technical replicates and statistically analyzed using Student’s *t*-test (^∗^*P* < 0.05, ^∗∗^*P* < 0.01). Bars indicate standard error of the mean. **(F)** The lesion area of the transgenic lines and (WT) were detected after 5 days of incubation with *P. sojae*. The experiments were performed on three biological replicates with their respective three technical replicates and statistically analyzed using Student’s *t*-test (^∗^*P* < 0.05, ^∗∗^*P* < 0.01). Bars indicate standard error of the mean. **(G)** The relative transcript abundance of *GmPR1a* (AF136636), *GmPR2* (M37753), *GmPR3* (AF202731), *GmPR4* (BT090788), *GmPR5a* (M21297), *GmPR10* (FJ960440) in *GmNPR1* overexpression transgenic lines (*GmNPR1***-**OE) and RNA-interference transgenic lines (*GmNPR1*-RNAi) were compared with that in WT. The amplification of *GmEF1b* gene (NM_001248778) was used as an internal control to normalize all the data. Statistically significant differences were performed between the transgenic lines and WT. The experiment was performed on three biological replicates with their respective three technical replicates and statistically analyzed using Student’s *t*-test (^∗^*P* < 0.05, ^∗∗^*P* < 0.01). Bars indicate standard error of the mean.

To determine whether the resistance to *P. sojae* phenotypes in *GmWRKY31* transgenic soybean plants is related to *GmNPR1* expression, we produced *GmNPR1*-overexpressing (*GmNPR1*-OE) and RNAi (*GmNPR1*-RNAi) transgenic plants. Southern blot results showed that three T2 *GmNPR1*-overexpressing transgenic lines (*GmNPR1*-OE) and three T2 *GmNPR1*-RNAi transgenic soybean lines were integrated into the genomes of the three transgenic lines with one copy, respectively (**Figure 7A**). These transgenic lines were developed into T3 transgenic soybean plants. Expression analysis of the transgenic population led to the identification of lines with remarkably increased *GmNPR1* mRNA levels, and the expression analysis of T3 *GmWRKY31*-RNAi transgenic soybean plants led to the identification of lines with significantly reduced *GmNPR1* mRNA levels (**Figure 7B**).

The living cotyledons of the *GmNPR1*-OE and *GmNPR1*-RNAi transgenic soybean plants were selected to investigate the resistance to *P. sojae*. After 2 days of incubation with zoospores of *P. sojae*, the remarkable differences in the development of disease symptoms were observed (**Figure 7C**). In *GmNPR1*-RNAi lines, the cotyledons became soft, emitted a foul odor and exhibited clear water-soaked lesions compared with those of the *GmNPR1*-OE lines. However, there were nearly no visible lesions in *GmNPR1*-OE lines, and the cotyledons keep hard compared with those of the *GmNPR1*-RNAi lines or WT. Meanwhile, the relative biomass of *P. sojae* in infected living cotyledons after 2 days of incubation with zoospores of *P. sojae* was also analyzed. The biomass of *P. sojae* based on the transcript level of the *P. sojae TEF1* gene was significantly (*P* < 0.01) lower in *GmNPR1*-OE lines than that in WT, and it was higher in *GmNPR1*-RNAi lines than that in WT but did not reach a significant level (**Figure 7D**). Similar results were obtained after 5 days of incubation with *P. sojae*, and the ability of living leaves to resist *P. sojae* significantly increased in the *GmNPR1*-OE lines and decreased in the *GmNPR1*-RNAi lines compared with WT (**Figure 7E**). The lesion area of the *GmNPR1*-OE lines was significantly (*P* < 0.01) smaller and the lesion area of the *GmNPR1*-RNAi lines was larger than that of WT after 5 days infection with *P. sojae* (**Figure 7F**). These results indicate that the overexpression of *GmNPR1* in soybean plants improved resistance to *P. sojae*, whereas susceptibility to *P. sojae* was enhanced in *GmNPR1*-RNAi transgenic soybean plants.

To determine whether GmNPR1 regulates *PR* gene expression, we analyzed mRNA levels in *GmNPR1* transgenic plants by qRT-PCR. *GmPR1a* (GenBank accession no. AF136636), *GmPR2* (GenBank accession no. M37753), *GmPR3* (GenBank accession no. AF202731), *GmPR4* (GenBank accession no. BT090788), *GmPR5a* (GenBank accession no. M21297), and *GmPR10* (GenBank accession no. FJ960440) were highly induced in *GmNPR1*-overexpressing lines, and the transcript abundance of *GmPR10* increased up to 12-fold compared to WT (**Figure 7G**). In *GmNPR1*-RNAi transgenic soybean plants, the transcript abundances of *GmPR1a*, *GmPR3*, *GmPR4*, *GmPR5a* and *GmPR10* were significantly lower than those of WT (**Figure 7G**). These data indicate that GmNPR1 directly or indirectly regulates these *PR* genes.

The T4 *GmNPR1* transgenic plants were identified by qRT-PCR (**Supplementary Figure S4A**), and the content of SA was analyzed in T4 *GmNPR1* transgenic plants. The results showed that the accumulation of SA in *GmNPR1*-OE lines was significantly (*P* < 0.05) higher than that in WT or *GmNPR1*-RNAi lines, and it was lower in *GmNPR1*-RNAi lines than that in WT but did not reach a significant level (**Supplementary Figure S4B**). These results indicated that overexpression of *GmNPR1* in soybean leads to higher SA level.

## Discussion

Since the first WRKY protein (SPF1) was identified in sweet potato (Rushton and Somssich, 1998), much progress has been made in studying the functions of WRKY family TFs. In model plants, the functional roles and underlying mechanisms of many WRKY TFs in pathogen responses have been studied (Chen and Chen, 2002; Kim et al., 2006; Chen et al., 2010; Abbruscato et al., 2012), but little is known about the functional roles of WRKYs and their mechanisms in soybean. In this study, we determined that *GmWRKY31*, a new member of the soybean WRKY family of TFs, plays a crucial role in soybean during infection with *P. sojae*. To cope with pathogenic challenge, plants rapidly activate defense responses regulated by the major signaling molecules SA, JA, and ET (Reymond and Farmer, 1998; Kunkel and Brooks, 2002; Spoel et al., 2003; Robert-Seilaniantz et al., 2011). *WRKY* genes are rapidly induced by pathogens, pathogen elicitors, or SA treatment in a number of plant species (Eulgem et al., 1999; Chen and Chen, 2000). Arabidopsis WRKY70 is a common regulatory component of SA- and JA- dependent defense responses and is also required for *R* gene-mediated resistance (Li et al., 2004, 2006; Knoth et al., 2007). In rice, OsWRKY13 functions as an activator of the SA-dependent defense response and a suppressor of the JA-dependent response, which mediates disease resistance to bacterial blight and fungal blast (Qiu et al., 2007). Here, we demonstrated that mRNA transcripts of *GmWRKY31* are remarkably increased by *P. sojae*, suggesting an important role of *GmWRKY31* in soybean resistance to *P. sojae*. To understand the molecular basis of GmWRKY31 in response to *P. sojae*, we further demonstrated that overexpression of *GmWRKY31* significantly increases resistance to *P. sojae* in transgenic soybean plants, whereas resistance to *P. sojae* was compromised in *GmWRKY31* RNA interference transgenic soybean plants. These results indicate that GmWRKY31 is required for soybean defense responses to *P. sojae*.

WRKY TFs are mainly involved in the response to biotic and abiotic stresses by binding to the W-box present in the promoter of defense-related genes (Maleck et al., 2000; Wang H.H. et al., 2015). OsWRKY4 specifically binds the promoter regions of *OsPR1b* and *OsPR5*, which contain W-box or W-box-like *cis*-elements that mediate resistance to rice sheath blight fungus (Wang H.H. et al., 2015). In this study, we demonstrated that GmWRKY31 is localized in the nucleus. We also demonstrated that GmWRKY31 activates basal transcription levels of a reporter gene in Arabidopsis cells. These findings suggest that GmWRKY31 acts as a W-box-mediated transcriptional activator. *WRKY* genes act upstream of *NPR1* and positively regulate its expression during the activation of plant defense responses (Yu et al., 2001). Liu et al. (2005) demonstrated that *OsNPR1* expression is constitutively induced in OsWRKY03 transgenic plants but not in control plants, suggesting that OsWRKY03 functions upstream of *OsNPR1* in the defense signal pathway. Accordingly, Choi et al. (2015) determined that OsWRKY6 positively regulates *OsNPR1* expression in transient expression assays but did not detect any amplified PCR bands by ChIP-PCR assay in *OsWRKY6*-ox lines, suggesting that OsWRKY6 does not directly bind to the *OsNPR1* promoter. We previously reported differential abundance of *GmNPR1* in response to the oomycete *P. sojae* by SSH coupled with cDNA microarrays (Xu et al., 2012). In this work, *GmWRKY31* overexpression activated *GmNPR1* gene expression, and *GmWRKY31* RNA interference transgenic soybean plants reduced *GmNPR1* gene expression. In addition, we also demonstrated that GmWRKY31 directly binds to the W-box within the -766 to -638 region of the *GmNPR1* promoter using EMSA and yeast-one-hybrid assays. Taken together, these results suggest that GmWRKY31 promotes *GmNPR1* expression by directly binding to the W-box within the -766 to -638 region of the *GmNPR1* promoter *in vivo* and *in vitro*.

WRKY TFs perform their diverse functions in various stress signaling pathways through physical interactions with different proteins, such as the VQ protein, MAP kinases, His deacetylases, and calmodulin (Park et al., 2005; Kim et al., 2008; Qiu et al., 2008; Ishihama et al., 2011). In Arabidopsis, AtWRKY33, which interacts with multiple VQ proteins, is critical not only for resistance to necrotrophic pathogens but also for tolerance to important abiotic stresses (Zheng et al., 2006; Jiang and Deyholos, 2009; Li et al., 2011). In rice, OsWRKY30 interacts with several rice MAPKs and is phosphorylated by some interacting MAPKs (Shen et al., 2012). More recent research indicates that GmWRKY27 interacts with GmMYB174, which cooperatively suppresses GmNAC29 expression and enhances drought stress tolerance in soybean plants (Wang F. et al., 2015). The present data demonstrate that GmWRKY31 interacts with GmHDL56 *in vivo* and *in vitro* and responds to *P. sojae* infection.

Homeodomain leucine-zipper (HD-ZIP) TFs are rapidly induced in response to altered environmental conditions (Brandt et al., 2014). GmHDL56 belongs to the HD-ZIP TF family and binds to the ATTAATTA sequence present in the promoter of *GmVspB* (Scott et al., 1989; Tang et al., 2001). The WRKY protein-binding elements and HD-ZIP protein-binding elements are located very close to each other in the promoter region of *GmNPR1*. We determined that *GmHDL56* overexpression activated *GmNPR1* gene expression and GmHDL56 also specifically bound to the ATTAATTA element (-1263 to -1205) present in the promoter of region of *GmNPR1*. We also demonstrated that GmWRKY31 and GmHDL56 cooperatively improved the expression of GmNPR1. These results suggested that the interaction between GmWRKY31 and GmHDL56 might contribute to the spatial and temporal patterns of expression of *GmNPR1*.

**FIGURE 8:**
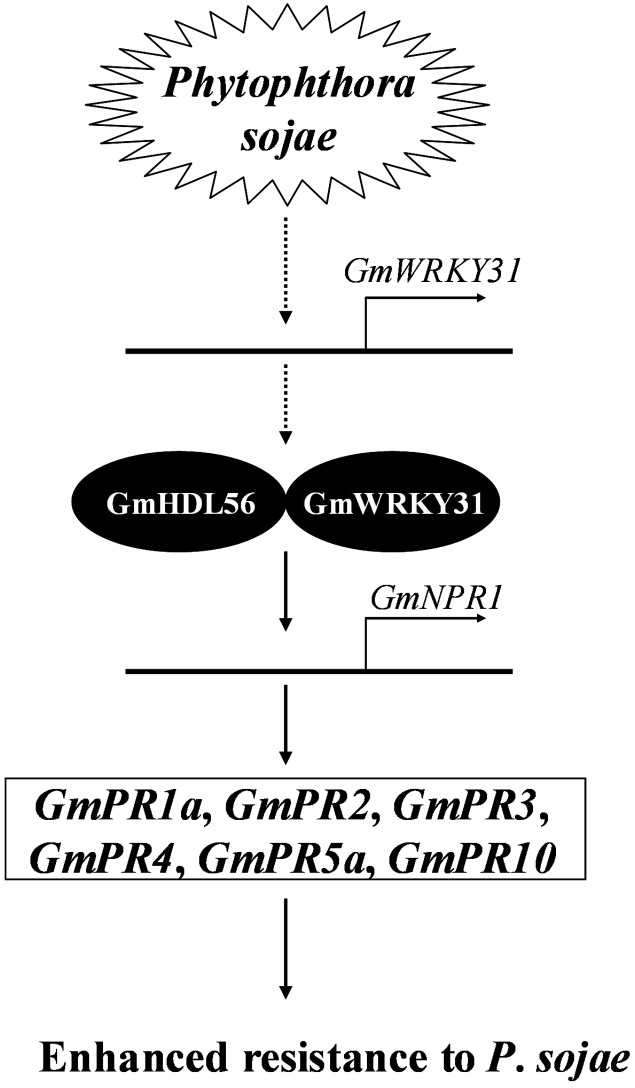
**Proposed model of GmWRKY31 function in response to *Phytophthora sojae* in soybean.** Expression of the *GmWRKY31* gene is induced by *P. sojae*. GmWRKY31 interacts with GmHDL56, and cooperatively activate the expression of *GmNPR1* by binding to the core sequences in its promoter. *GmNPR1* positively regulates the expression of the *GmPR1a* (AF136636), *GmPR2* (M37753), *GmPR3* (AF202731), *GmPR4* (BT090788), *GmPR5a* (M21297), *GmPR10* (FJ960440) genes, finally leading to enhance resistance to *P. sojae* in soybean.

NPR1 was first identified by screening for mutants blocked in SA-mediated *PR* gene expression and resistance (Cao et al., 1994; Delaney et al., 1995; Wang et al., 2006). *NPR1* is constitutively expressed in plants, and its expression level is elevated upon SA treatment or pathogen infection (Malnoy et al., 2007; Shi et al., 2010; Zhang et al., 2012). In addition, the overexpression of the *AtNPR1* gene or its orthologs enhances resistance to biotrophic and necrotrophic fungal, viral and bacterial pathogens in a number of plant species, including rice, wheat, tomato and tobacco (Lin et al., 2004; Chern et al., 2005; Makandar et al., 2006; Yuan et al., 2007; Meur et al., 2008). In the present study, the expression of *GmNPR1* was significantly induced by *P. sojae*. Further transgenic analysis revealed that the overexpression of *GmNPR1* in soybean improved resistance to *P. sojae* and enhanced susceptibility to *P. sojae* infection in *GmNPR1*-RNAi lines, and the accumulation of SA in the *GmNPR1*-OE lines was significantly higher than that of WT and *GmNPR1*-RNAi lines. In Arabidopsis, the TF NPR1 is essential for the induction of defense genes such as PR genes (Zhou et al., 2000; Fan and Dong, 2002). Here, we observed that *GmPR1a*, *GmPR2*, *GmPR3*, *GmPR4*, *GmPR5a*, and *GmPR10* were highly induced in *GmNPR1*-OE lines and that *GmPR1a*, *GmPR3*, *GmPR4*, *GmPR5a* and *GmPR10* were significantly repressed in *GmNPR1*-RNAi lines. Taken together, these results indicated that overexpression of *GmNPR1* in soybean leads to higher SA level. Previous studies with SA in other plants showed that SA-induced defense responses are mediated by NPR1 (Cao et al., 1997; Spoel et al., 2003) and the accumulation of SA leads to up-regulation of defense-related genes including the PR genes *PR1*, *PR2* and *PR5*, and results in enhanced disease resistance against biotrophic pathogens (Gaffney et al., 1993; Delaney et al., 1994). So, we speculate a model that GmWRKY31 and GmHDL56 interact with each other and directly activate the expression of *GmNPR1*, then GmNPR1 positively regulates the content of the SA, and accumulation of SA leads to up-regulation of *GmPR1a*, *GmPR2*, *GmPR3*, *GmPR4*, *GmPR5a*, and *GmPR10* genes, which ultimately leads to enhanced resistance to *P. sojae* in soybean (**Figure 8**).

In addition, the *GmNPR1*-OE lines show a 15–20 fold increase in transcripts for *GmNPR1*, but the GmWRKY31 or GmHDL56 transactivation assays show a rather weak increase in *GmNPR1* (<2-fold). In previous studies, there were reports that a few of genes were induced by *P. sojae*, such as N-rich protein (Ludwig and Tenhaken, 2001), *GmPR10* (Xu et al., 2014), *GmPRP* (Jiang et al., 2015), and *Gly m 4l* (Fan et al., 2015), then we speculate that there maybe *P. sojae* induced genes changed in their expression when the expression of GmWRKY31 or GmHDL56 is modified, which might affect the expression of *GmNPR1*. So, in the follow-up work, it is valuable to make a deeper research on other *P. sojae* induced genes, defensive barriers and phytoalexins in transgenic soybean plants, and it would improve the interpretation of the findings that the mechanism of the GmWRKY31 and GmHDL56 enhances resistance. This study provides new insights into the mechanism by which WRKY proteins regulate biotic stress responses in soybean and presents GmWRKY31 as an ideal candidate target to improve *P. sojae* resistance in soybean and other crops.

## Author Contributions

Conceived and designed the experiments: PX and SZ. Performed the experiments: SF, LD, DH, and FZ. Analyzed the data: JW, LJ, WL, QC, RL, and FM. Contributed reagents/materials/analysis tools: SZ and PX.

## Conflict of Interest Statement

The authors declare that the research was conducted in the absence of any commercial or financial relationships that could be construed as a potential conflict of interest.

## Footnotes

1 http://www.phytozome.net/

2 http://www.cambia.org

3 http://www.clontech.com

4 http://blast.ncbi.nlm.nih.gov/Blast.cgi

## Supplementary Material

FIGURE S1 **Sequence and sequence alignment of *GmWRKY31* gene. (A)** The open reading frame sequence and deduced polypeptide sequence of *GmWRKY31*. **(B)** Phylogenetic relationship between GmWRKY31 and the related WRKY proteins in other species. **(C)** Alignment of the conserved WRKY domain amino acid sequences of GmWRKY31 and nearby 7 WRKY proteins in **(B)**.

FIGURE S2 **Expression patterns of *GmWRKY31* in soybean. (A)** *GmWRKY31* mRNA levels in various tissues of soybean. **(B)** *GmWRKY31* expression in soybean leaves infected with *Phytophthora sojae*. **(C)** *GmWRKY31* expression in soybean resistant cultivars and susceptible cultivars.

FIGURE S3 **Expression patterns of *GmNPR1* in soybean. (A)** *GmNPR1* mRNA levels in various tissues of soybean. **(B)** *GmNPR1* expression in soybean leaves infected with *Phytophthora sojae*. **(C)** *GmNPR1* expression in soybean resistant cultivars and susceptible cultivars.

FIGURE S4 **The content of SA in T_4_*GmNPR1* transgenic plants. (A)** Quantitative real-time PCR of the T4 *GmNPR1*-overexpression. **(B)** The content of SA in T_4_ *GmNPR1* transgenic plants.
