# Supplementary figures and images for: GmWRKY31 and GmHDL56 Enhances Resistance to Phytophthora sojae by Regulating Defense-Related Gene Expression in Soybean
Source: Front Plant Sci. 2017 May 12;8:781. doi: 10.3389/fpls.2017.00781 (PMC5427154; doi:10.3389/fpls.2017.00781)

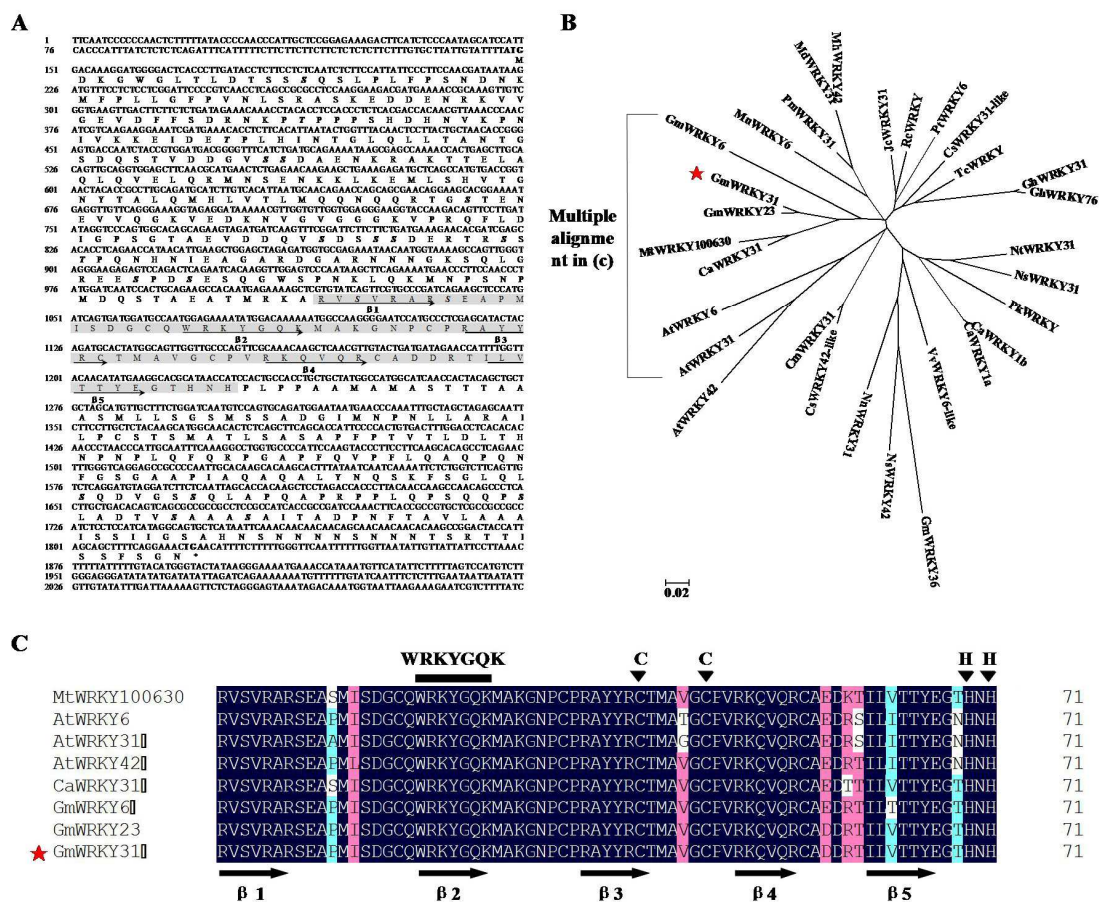

Supplementary Figure 1

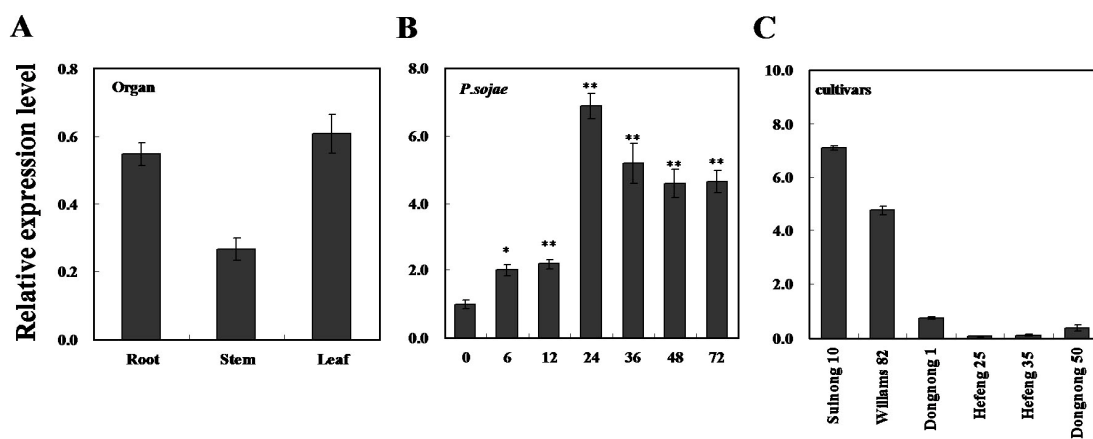

Supplementary Figure 2

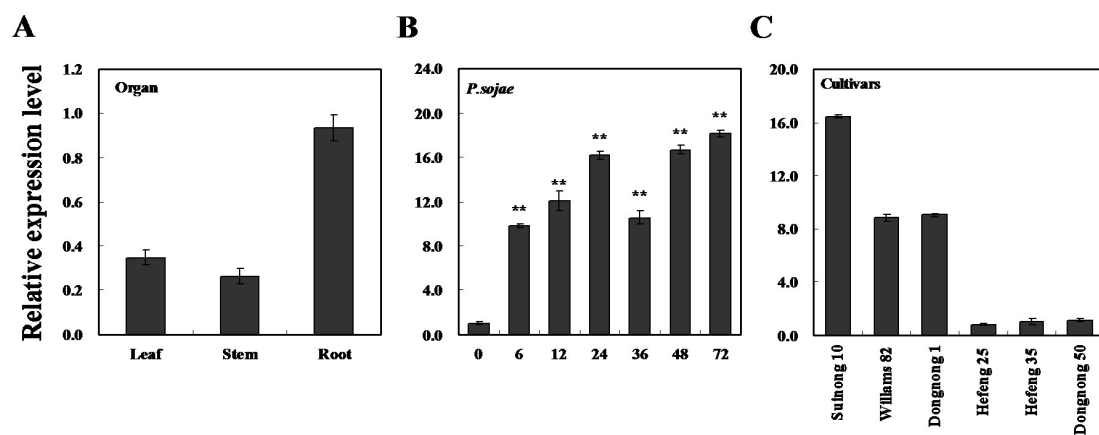

Supplementary Figure 3

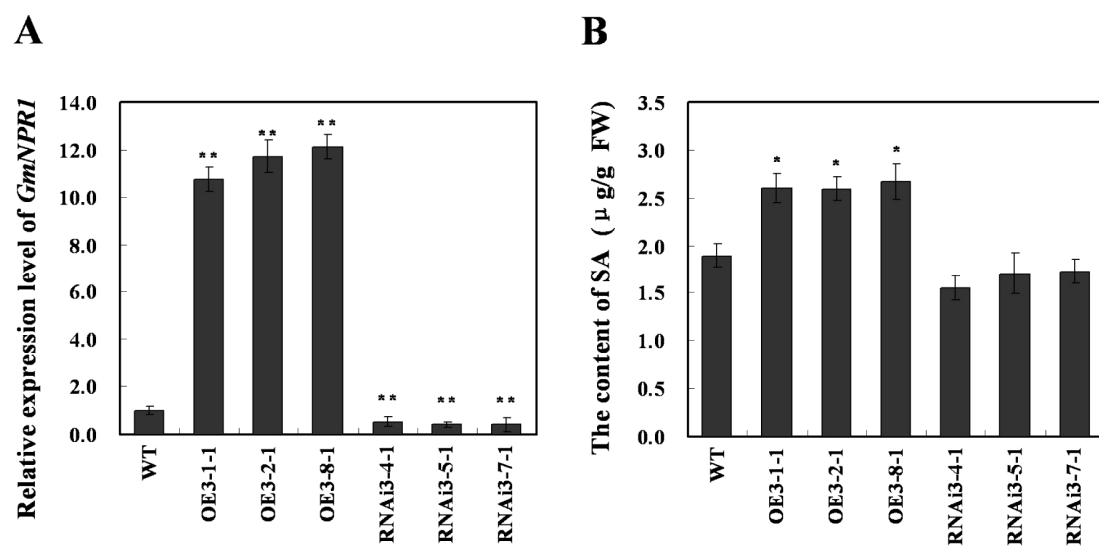

Supplementary Figure 4

Supplement: FIGURE S1 — Sequence and sequence alignment of GmWRKY31 gene. (A) The open reading frame sequence and deduced polypeptide sequence of GmWRKY31. (B) Phylogenetic relationship between GmWRKY31 and the related WRKY proteins in other species. (C) Alignment of the conserved WRKY domain amino acid sequences of GmWRKY31 and nearby 7 WRKY proteins in (B). [file Image_1.PDF]
