# Supplementary material for: GmWRKY31 and GmHDL56 Enhances Resistance to Phytophthora sojae by Regulating Defense-Related Gene Expression in Soybean
Source: Front Plant Sci. 2017 May 12;8:781. doi: 10.3389/fpls.2017.00781 (PMC5427154; doi:10.3389/fpls.2017.00781)
Supplement: Supplementary file 2 [file Table_1.DOC]

**Supplementary Table S1.** Oligonucleotide primers used in this study.

| **Overexpression** | *GmWRKY31*-*F* | GAAGATCTAATGGACAAAGGATGGGGACTC |
| --- | --- | --- |
|  | *GmWRKY31*-*R* | GGGTAACCTCAGTTTCCTGAAAAGCTGCTA |
|  | *GmNPR1*-*F* | GAAGATCTAATGGCTTATTCAGCCGAAC |
|  | *GmNPR1*-*R* | GGGTAACCTTACACTTTCCTAGCCTTGTAAT |
|  | *GmHDL56*-*F* | GAAGATCTATGAAGAGACTTGGCAGTT |
|  | *GmHDL56*-*R* | GCACGTGACTCCATTCCTCTGAACAGTA |
|  | *Bar*-*F* | TTTCAATCCTAATAACAACGAGC |
|  | *Bar*-*R* | ATGGACAACAACAGCCCTTC |
| **qRT-PCR** | *GmWRKY31*-*qF* | ACCTCACACACAACCCTAACCC |
|  | *GmWRKY31*-*qR* | CCTACATCCTGAGACAACTGAAGAC |
|  | *GmEF1b*-*qF* | CCACTGCTGAAGAAGATGATGATG |
|  | *GmEF1b*-*qR* | AAGGACAGAAGACTTGCCACTC |
|  | *GmNPR1*-*qF* | TCTTTGGGTTTTCGGTCT |
|  | *GmNPR1*-*qR* | CAACTTTCCTGCTTTCACA |
|  | *TEF1*-*qF* | TGATCGTGCTGAACCACCC |
|  | *TEF1*-*qR* | CGAGCGACGGTCCATCTT |
|  | *GmPR1a*-*F* | TGAAAATGTGGGTTGATGAGAAAT |
|  | *GmPR1a*-*R* | AAGTGATGAAAGTGCCTCCGTT |
|  | *GmPR2*-*F* | GTTCGGAATGTGAAGCAAGGA |
|  | *GmPR2*-*R* | ATAGGAGAAAAGAGCCGCCAA |
|  | *GmPR3*-*F* | AACACCAACTCCAACAACACCTA |
|  | *GmPR3*-*R* | TGCCAAAGCCATTGAAAGA |
|  | *GmPR4*-*F* | AACGCCGTGAGTGCTTATTGT |
|  | *GmPR4*-*R* | TTTGCTCCTGTTCCTGTATTTGTC |
|  | *GmPR5a*-*F* | CCCTCGCCTCCACTTCTTC |
|  | *GmPR5a*-*R* | TTGGTGCTCATCTTGCCTCTA |
|  | *GmPR10*-*F* | TAGCATCCACAGCATTGTTTTC |
|  | *GmPR10*-*R* | CAAGGCAGTGCCCTCAGTTA |
| **GFP** | *GmWRKY31*-*gF* | GGAAGATCTTATGGACAAAGGATGGGGACTC |
|  | *GmWRKY31*-*gR* | CGGACTAGTGTTTCCTGAAAAGCTGCTAATGG |
| **EMSA and Pull down** | *GmWRKY31*-*yF* | CGGGATCCTATGGACAAAGGATGGGGACTC |
|  | *GmWRKY31*-*yR* | CCGGAATTCGGGTTTCCTGAAAAGCTGCTAAT |
|  | *GmHDL56-yF* | GAAGATCTAATGAAGAGACTTGGCAGTTCT |
|  | *GmHDL56-yR* | GCGTCGACACTCCATTCCTCTGAACAGTAC |
| **Yeast one-hybrid** | *GmWRKY31-oF* | CCGGAATTCATGGACAAAGGATGGGGACTC |
|  | *GmWRKY31-oR* | CGGGATCCGTTTCCTGAAAAGCTGCTAATGG |
|  | *N1-F* | CCGGAATTCTTGACCTTCACAGCCGC |
|  | *N1*-*R* | CGAGCTCAGTCAAAGCATCATAAACAGG |
|  | *mN1*-*F* | CCGGAATTCTcGAtCTTCACAGCCGC |
|  | *mN1*-*R* | CGAGCTCAaTCgAAGCATCATAAACAGG |
|  | *GmHDL56-oF* | CCGGAATTCATGAAGAGACTTGGCAGTTCTG |
|  | *GmHDL56-oR* | CGGGATCCACTCCATTCCTCTGAACAGTAC |
| **Promoter cloning** | *GmWRKY31*-*pF* | CCCAAGCTTCAAAGACTAAGTTACTTGGGCA |
|  | *GmWRKY31*-*pR* | CGGGATCCAAAATACAATAAGCACAAAGAAG |
| **RNAi** | *GmWRKY31*-*rF* | GGGGACAAGTTTGTACAAAAAAGCAGGCTCAGCGAACAGGAAGCACG |
|  | *GmWRKY31*-*rR* | GGGGACCACTTTGTACAAGAAAGCTGGGTGGCACGAACTGATACACG |
|  | *GmNPR1*-*rF* | GGGGACAAGTTTGTACAAAAAAGCAGGCTCGCTAATGTCAATCTTCG |
|  | *GmNPR1*-*rR* | GGGGACCACTTTGTACAAGAAAGCTGGGTGCAAATGCCACTCTGTTC |
|  | *Pat*-*F* | CCGGCAACAATTAATAGACT |
|  | *Pat*-*R* | TCCATAGTTGCCTGACTCCC |
| **Yeast two-hybrid** | *GmWRKY31-tF* | GCCGAATTCATGGACAAAGGATGGGGACTC |
|  | *GmWRKY31-tR* | GGTCGACGTTTCCTGAAAAGCTGCTAATGG |
|  | *GmHDL56-tF* | CCGGAATTCATGAAGAGACTTGGCAGTTCTG |
|  | *GmHDL56-tR* | CGCGGATCCACTCCATTCCTCTGAACAGTAC |
| **BIFC** | *GmWRKY31-bF* | CCGGAATTCATGGACAAAGGATGGGG |
|  | *GmWRKY31-bR* | CGCGGATCCAGTTTCCTGAAAAGCTG |
|  | *GmHDL56-bF* | CCGGAATTCATGAAGAGACTTGGCAGTTCTG |
|  | *GmHDL56-bR* | CGCGGATCCCACTCCATTCCTCTGAACAGTAC |
