# Supplementary material for: GmWRKY31 and GmHDL56 Enhances Resistance to Phytophthora sojae by Regulating Defense-Related Gene Expression in Soybean
Source: Front Plant Sci. 2017 May 12;8:781. doi: 10.3389/fpls.2017.00781 (PMC5427154; doi:10.3389/fpls.2017.00781)
Supplement: Supplementary file 3 [file Table_2.DOC]

**Supplementary** **Table S2.** Part of positive colonies were identified using the homology analysis BLAST in NCBI.

| Protein | Gene ID | Colonies |
| --- | --- | --- |
| Synaptotagmin-2-like (*SYT2L*) | 100778906 | 36 |
| Ras-related protein RABE1c-like (*RABE1c*) | 100779203 | 6 |
| Thylakoid membrane phosphoprotein 14 kDa, chloroplastic-like | 100793015 | 6 |
| Chromo domain-containing protein LHP1-like (*LHP1*) | 100812329 | 4 |
| Glycine-rich RNA-binding protein (*GRP*) | 547560 | 4 |
| SNF-1-like serinethreonine protein kinase (*SnRK*) | 547544 | 3 |
| Dirigent protein 3-like (*DIR3*) | 100807466 | 3 |
| Zinc finger CCCH domain-containing protein ZFN-like (*ZFN*) | 100802536 | 3 |
| psbP domain-containing protein 4 | 100800838 | 3 |
| F-box protein SKIP14-like (*SKIP14*) | 100778863 | 2 |
| Ubiquitin-conjugating enzyme E2 7-like (*UBCE2-7*) | 100782328 | 2 |
| Light-inducible protein CPRF2-like (*CPRF2*) | 100798673 | 2 |
| Aspartic proteinase oryzasin-1-like (*APs*) | 100796053 | 2 |
| DNA-damage-repair/toleration protein DRT100-like (*DRT100*) | 100814412 | 2 |
| Eukaryotic translation initiation factor 1A-like (*EIF*) | 100818207 | 1 |
| Proline-rich protein (*SBPRP*) | 547594 | 1 |
| homeodomain-leucine zipper protein 56 (*HDL56*) | 100796213 | 1 |
| bZIP transcription factor bZIP105 (*bZIP105*) | 778137 | 1 |
| Glycogen synthase-like | 100776823 | 1 |
